# Ectomycorrhizal Fungi Associated with *Pinus cembroides* subsp. *orizabensis*, an Endemic Pine in the Arid Zones of the Oriental Basin, Puebla, Mexico

**DOI:** 10.3390/jof11090677

**Published:** 2025-09-16

**Authors:** Yajaira Baeza-Guzmán, Marian Silvana Vásquez-Jiménez, Elvira Morgado-Viveros, Luz Amelia Sánchez-Landero, Dora Trejo-Aguilar

**Affiliations:** 1 Facultad de Ciencias Agrícolas, Universidad Veracruzana, Zona Universitaria, s/n, Xalapa 91000, Mexico; ybaeza@uv.mx (Y.B.-G.); luzsanchez02@uv.mx (L.A.S.-L.); 2 Facultad de Biología, Universidad Veracruzana, Zona Universitaria, s/n, Xalapa 91000, Mexico; mariansilvana@gmail.com (M.S.V.-J.); elmorvi@hotmail.com (E.M.-V.)

**Keywords:** cover tree, ectomycorrhizal symbiosis, erosion, fungal diversity, pinyon pine

## Abstract

Ectomycorrhizal fungi (EMF) associated with the roots of *Pinus cembroides* subsp. *orizabensis*, a key pinyon pine species for local forestry in the Oriental Basin, Puebla, Mexico, were identified and analyzed. The study aimed to evaluate the diversity of EMF in this endemic pine across three sampling transects (T1, T2, T3), each located in sites with different vegetation compositions and pine cover. In each site, a 100 m × 25 m transect was established, and root tips colonized by EMF were collected for morphological and molecular identification. Alpha (α) and beta (β) diversity were calculated for each transect. A total of 16 EMF morphotypes were identified, and molecular analysis confirmed four taxa: *Geopora arenicola*, *Rhizopogon* aff. *subpurpurascens*, *Tomentella* sp. 1, and *Tricholoma* sp. 1. The transect with the highest *P. cembroides* cover showed the greatest fungal richness. Beta diversity, as measured by Sørensen index partitioning, revealed a 30% species turnover between T1 and T2 and a 60% turnover between T2 and T3, suggesting distinct fungal communities. In contrast, no turnover but a nested pattern was observed between T1 and T3, indicating that the less diverse community is a subset of the richer one. These results show that EMF composition varies with pine cover and vegetation heterogeneity, highlighting the influence of disturbance on fungal diversity. This is the first report of EMF fungi associated with *Pinus cembroides* subsp. *orizabensis*, as well as the first record of *G. arenicola* in arid pine forests in Mexico.

## 1. Introduction

Ectomycorrhizal symbiosis is fundamental for the survival and growth of pine species, particularly in harsh environments where water and nutrient availability is limited [1]. Ectomycorrhizal fungi (EMF) play a crucial role by facilitating the uptake of essential nutrients such as nitrogen and phosphorus through the extension of their mycelium in the soil, thereby increasing the absorptive surface area and nutrient availability for the host plant. They also enhance drought tolerance by improving water retention in the rhizosphere and increasing water-use efficiency in roots. Additionally, EMF protect plants from soil pathogens by competing for space and resources and by stimulating plant defenses via primary and secondary metabolism [2].

In species adapted to semi-arid environments, such as *Pinus cembroides* subsp. *orizabensis* [3], these symbiotic associations are critical for establishment and persistence in degraded ecosystems, such as eroded or nutrient-poor soils, where environmental stress limits plant survival and development [4]. Globally, arid and semi-arid regions represent more than 40% of the Earth’s terrestrial surface and are expanding due to climate change and land degradation [5]. In these areas, EMF play a pivotal role in supporting vegetation resilience. Studies conducted in China, United States, and Mediterranean and African drylands have shown that EMF diversity is closely linked to ecosystem stability and plant community structure under water-limited conditions [6,7,8,9]. Thus, advancing our understanding of EMF associations in arid zones has broad implications for global restoration strategies. *Pinus cembroides* subsp. *orizabensis* is an endemic Mexican subspecies adapted to rocky soils and cool, slightly humid climates. Among the pinyon pines, it has the southernmost distribution, primarily occurring in the states of Puebla, Tlaxcala, and Veracruz [10,11]. It is highly resilient to extreme climatic conditions and is capable of growing in both shallow and deep soils, with a pH range of four to eight, making it an excellent candidate for reforestation in areas with low rainfall or eroded soils [12].

Despite its ecological importance and reforestation potential, conservation efforts have focused mainly on phenotypic traits and environmental requirements such as soil properties [12,13].

Key aspects like its symbiotic relationship with EMF have been largely overlooked in restoration programs. Moreover, land-use change poses a growing threat to this subspecies, as the expansion of agriculture, rising temperatures, and reduced water availability diminish both the quality and extent of its natural habitat, potentially impacting its long-term distribution and survival [14,15]. These environmental changes contribute to degraded soils, increased water stress, and the loss of critical microhabitats necessary for plant development.

Reforestation efforts have shown limited success, with low establishment rates and high mortality among reintroduced individuals, likely due to suboptimal soil and climate conditions and poor genotypic adaptation to the new planting areas. Previous studies have shown that EMF richness and diversity tend to decline in highly disturbed environments [16] which may partly explain the low success rates of reforestation programs involving this species [17].

Identifying the EMF communities associated with *Pinus cembroides* subsp. *orizabensis* is essential for designing effective restoration strategies. This study aimed to evaluate the alpha (α) and beta (β) diversity of EMF associated with this subspecies. Morphological characterization and DNA sequencing methods were used to identify ectomycorrhizal morphotypes from root samples. The results will contribute to understanding EMF ecology in *P. cembroides* subsp. *orizabensis* under arid and semi-arid conditions and provide a scientific basis for incorporating native EMF as biofertilizers to improve survival and growth rates in reforestation efforts.

## 2. Materials and Methods

### 2.1. Study Area

The study was conducted in the municipality of Tepeyahualco, Puebla, within the geological depression known as the Oriental Basin (19°29′32.04″ N, 97°30′37.17″ W). This region contains remnants of *Pinus cembroides* subsp. *orizabensis* forests, xerophytic shrubland, and grasslands. The climate is predominantly temperate sub-humid, with annual precipitation below 300 mm and temperatures ranging from 12 to 16 °C. The soils are sandy and highly stony, primarily classified as Regosols and Leptosols [18].

### 2.2. Sampling Design

Three transects, each 100 m long and 25 m wide, were established in a north–south orientation. The transects were spaced 45 m apart and located at elevations of 2371, 2395, and 2409 m above sea level, respectively. Within each transect, five *P. cembroides* subsp. *orizabensis* trees with a diameter at breast height between 60 and 70 cm was randomly selected, and their mycorrhizal roots were collected.

Using photointerpretation of a Google Earth image and Quantum GIS software version 3.16, the canopies of *Pinus cembroides* subsp. *orizabensis* were identified to verify which transect had the greatest coverage of this species.

Soil samples were collected by removing the surface organic layer and boring to a depth of 25–30 cm adjacent to the base of each selected tree at the four cardinal points. Samples were placed in labeled Ziploc bags and transported under cool, shaded conditions to avoid direct sunlight and temperature fluctuations. Laboratory processing followed the modified protocol of Baeza-Guzmán et al. [19], adapted to the site’s edaphic conditions.

### 2.3. Vegetation Characterization of the Transects

**Figure 1:**
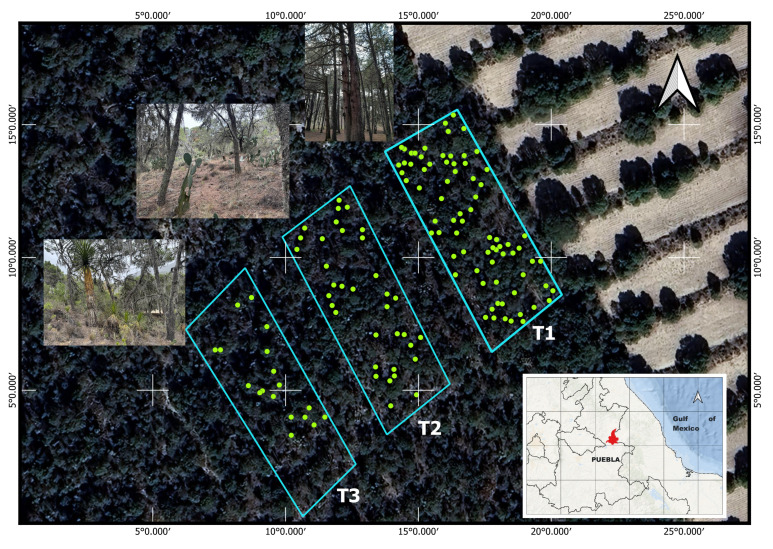
Location of the study area and transects (T1, T2, and T3). Green points correspond to *Pinus cembroides* subsp. *orizabensis* trees.

Transect T1 exhibited the highest vegetation cover of *P. cembroides* subsp. *orizabensis* and the lowest presence of understory species. Transect T2 showed a more heterogeneous plant composition than T1, although *P. cembroides* subsp. *orizabensis* remained the predominant forest vegetation among isolated individuals of *Juniperus deppeana*. A higher presence of understory species such as *Yucca periculosa*, *Agave obscura*, and *A. gilbeyi* was observed compared to T1. Transect T3 had the lowest cover of *P. cembroides* subsp. *orizabensis* among the three transects and showed evidence of logging of this species. It was dominated by *Yucca periculosa* and contained the highest presence of understory species such as *Mimosa biuncifera*, *Opuntia streptacantha*, *Opuntia* sp., *Muhlenbergia robusta*, and *Agave obscura* (Figure 1). Vegetation characterization was based on the analysis by Granados-Victorino et al. [12].

### 2.4. Sample Processing and Taxonomic/Molecular Root Tip Identification

To remove soil and organic debris, root and soil samples from each tree were homogenized and washed through a 2 mm mesh sieve. Mycorrhizal roots were then rinsed with distilled water using a 1 mm mesh sieve. A Velab VE-S0 stereomicroscope was used to dissect and distinguish hydrated, necrotic, and dead roots. Each mycorrhizal root tip was photographed and quantified for absolute and relative abundance per transect. Morphological identification of mycorrhizal roots was conducted based on branching type, presence of emanating hyphae or rhizomorphs, length of the mycorrhizal system, tip shape, texture, color, and mantle surface characteristics, following the criteria of Agerer and Rambold [20].

Additionally, taxonomic identification was performed through DNA extraction. Root morphotypes were preserved in liquid nitrogen and stored at −20 °C until DNA extraction using the DNeasy PowerSoil^®^ Kit (Qiagen, Hilden, Germany). Amplification of the internal transcribed spacer region (ITS2) and conserved regions of 5.8S and 28S rRNA genes was performed using the universal primers ITS3 (5′-GCATCGATGAAGACGCAGC-3′) and ITS4 (5′-TCCTCCGCTTATTGATATGC-3′) [21]. The PCR conditions included an initial denaturation at 94 °C for 5 min, followed by 35 cycles of 94 °C for 50 s, 58 °C for 50 s, and 72 °C for 50 s, with a final extension at 72 °C for 10 min. PCR products were purified and sequenced by Macrogen Inc. (Seoul, Republic of Korea; https://dna.macrogen.com). Sequence editing and alignment were conducted using Geneious Prime software, version 2023.2. Operational Taxonomic Units (OTUs) were defined at 97% similarity, and consensus sequences were compared against UNITE and GenBank (NCBI) databases.

### 2.5. Statistical and Diversity Analyses

Sampling completeness was assessed to determine whether the sampling effort was sufficient to capture the fungal diversity in the study area, based on the definition of completeness as the proportion of individuals in the community represented by the observed species [22]. Fungal morphotype diversity was evaluated using Hill numbers of different diversity orders (q = 0, 1, 2), which quantify species richness and evenness in fungal communities [23]. Both sampling completeness and diversity analyses were performed using the iNEXT package in R [24].

## 3. Results

### 3.1. Morphotype Richness per Transect

**Figure 2:**
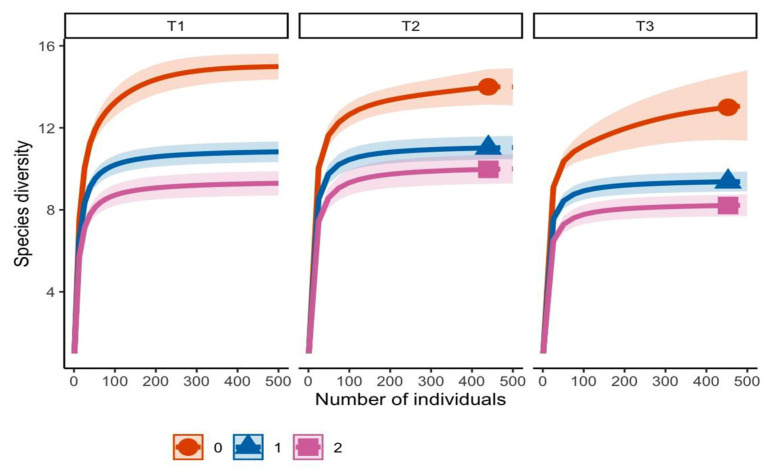
Hill numbers analysis for the ectomycorrhizal (EMF) morphotype community associated with roots of *P. cembroides* subsp. *orizabensis*. T1 (transect 1), T2 (transect 2), T3 (transect 3). Orange line (q0 = richness), blue line (q1 = Shannon diversity), pink line (q2 = Gini–Simpson diversity).

A total of 701 mycorrhizal root tips in T1 were classified into 16 morphotypes; transect T2 had 511 tips characterized into 15 morphotypes, and transect T3 had 558 tips characterized into 14 morphotypes. Morphotype richness (q0) was highest in T1, followed by T2 and T3. Shannon diversity (q1), which accounts for morphotype evenness, was also highest in T1, while T2 and T3 exhibited lower uniformity in morphotype distribution. Simpson diversity (q2), emphasizing dominant morphotypes, showed less marked differences among sites, suggesting that T2 and T3 communities were dominated by few morphotypes. Overall, the results indicate that transect 1 hosted the most diverse and balanced community, whereas T2 and T3 had lower species richness and evenness (Figure 2).

### 3.2. Sampling Completeness Analysis

**Figure 3:**
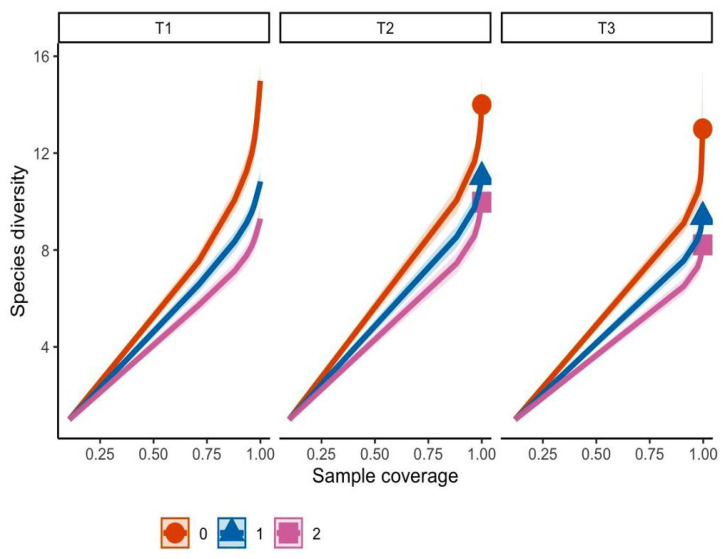
Sampling completeness analysis. T1 (transect 1), T2 (transect 2), T3 (transect 3). Orange line (q0 = richness), blue line (q1 = Shannon diversity), pink line (q2 = Gini–Simpson diversity).

The sampling completeness analysis (Figure 3) demonstrated that morphotype diversity increased as the sample coverage approached 1, indicating that the sampled proportion adequately represented the fungal community in the study area. Transect T1 contained a more diverse community regardless of sampling effort compared to the other two sites.

### 3.3. Beta Diversity Analysis

**Figure 4:**
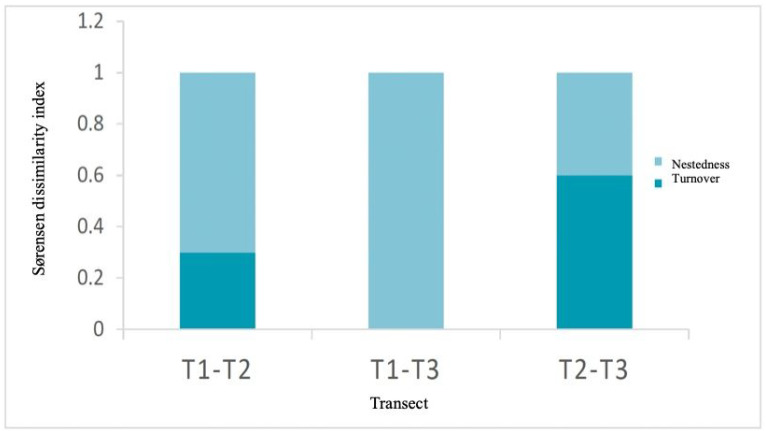
Sørensen dissimilarity index (βSOR) and its components: turnover (βSIM) and nestedness (βSNE) of the EMF morphotypes present in the three transects.

Beta diversity analysis (Figure 4) among transects was mostly explained by the nestedness component. Between transects T1 and T2, species turnover was 30%, indicating partial replacement of fungal species, possibly influenced by the greater tree canopy cover in T1, which also had the highest canopy cover and lower understory presence. In contrast, between T1 and T3, a pattern of total nestedness without turnover was observed, suggesting that the transect with lower diversity contained only a subset of species present in the more diverse transect. The greatest dissimilarity was found between T2 and T3, with a 60% turnover, indicating very distinct fungal communities, likely related to differences in vegetation composition.

### 3.4. Characterization of EMF Morphotypes

**Figure 5:**
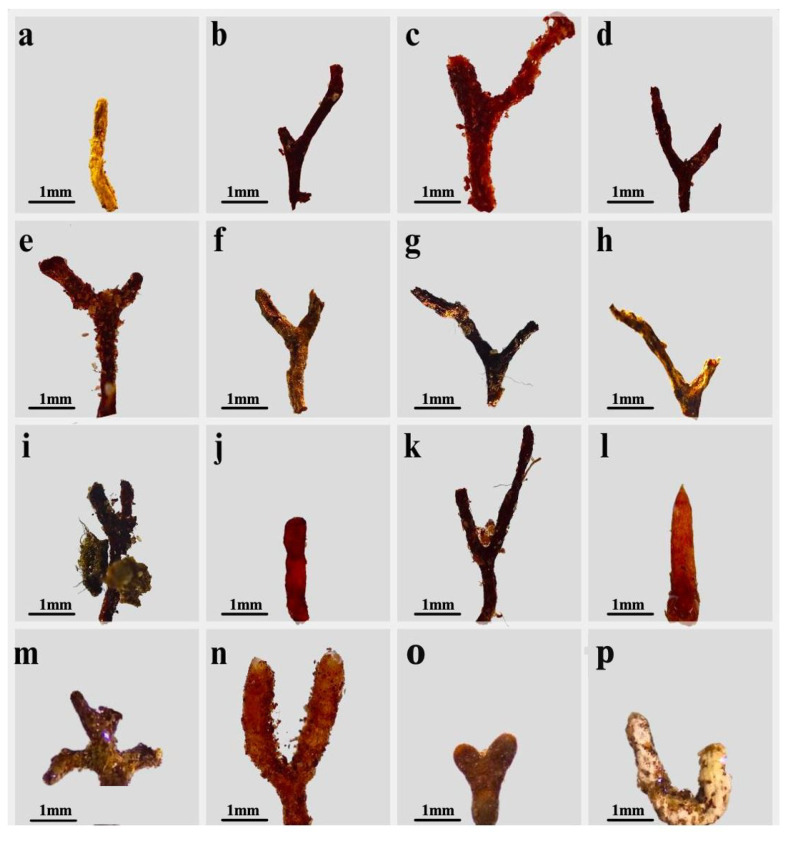
Ectomycorrhizal morphotypes. (**a**) *Inocybe* sp. 1 (morphotype M1); (**b**) *Pinirhiza* sp. 1 (morphotype M2); (**c**) *Pinirhiza* sp. 2 (morphotype M3); (**d**) *Tomentella* sp. 1 (morphotype M4); (**e**) *Pinirhiza* sp. 3 (morphotype M5); (**f**) *Rhizopogon aff. subpurpurascens* (morphotype M6); (**g**) *Pinirhiza* sp. 4 (morphotype M7); (**h**) *Tricholoma* sp. 1 (morphotype M8); (**i**) *Cenococcum* sp. 1 (morphotype M9); (**j**) *Geopora arenicola* (morphotype M10); (**k**) *Bankera* sp. 1 (morphotype M11); (**l**) *Russula* sp. 1 (morphotype M12); (**m**) *Amphinema* sp. (morphotype M13); (**n**) *Tylospora* sp. (morphotype M14); (**o**) *Russulaceae* (morphotype M15]); (**p**) *Paxillus* sp. (morphotype M16).

A total of 16 ectomycorrhizal (EMF) morphotypes associated with roots of *P. cembroides* subsp. *orizabensis* were characterized taxonomically within eight genera of the phylum Basidiomycota (*Amphinema*, *Bankera*, *Inocybe*, *Paxillus*, *Russula*, *Tomentella*, *Tricholoma*, *Tylospora*) and three genera of the phylum Ascomycota (*Cenococcum*, *Geopora*, *Rhizopogon*) (Table 1, Figure 5). Six morphotypes were analyzed for genetic identification, four of which matched information available in the UNITE and GenBank (NCBI) databases, while two morphotypes lacked conclusive data for taxonomic identification.

### 3.5. Genetic Identification of EMF Morphotypes

Six of the sixteen characterized morphotypes were identified through ITS region analysis. Despite DNA extraction attempts for the remaining 10 morphotypes, low DNA quality and concentration prevented obtaining high-quality sequences. This limitation should be considered in future studies, as low precipitation, soil type, and root depth influenced the ability to obtain turgid root tips. Four Operational Taxonomic Units (OTUs) matched database entries: *Tricholoma* sp. 1, *Tomentella* sp. 1, *Rhizopogon* aff. *subpurpurascens*, and *Geopora arenicola*. The remaining two morphotypes showed no matches in the referenced databases (Table 2).

## 4. Discussion

This study represents the first report on the native community of ectomycorrhizal fungi (EMF) associated with *Pinus cembroides* subsp. *orizabensis*, an endemic species of Mexico with high economic value due to the commercialization of its pine nuts, a nutritious and traditional food product that provides important income for rural communities and supports sustainable forest conservation [25]. *Rhizopogon* aff. *subpurpurascens* was identified as the most abundant species in transects T1 and T2.

Although 16 distinct EMF morphotypes were morphologically characterized, only 6 were successfully identified using molecular methods. This limitation primarily was due to the poor amplification of fungal DNA in some samples, likely caused by low-quality DNA from degraded or heavily melanized root tips [26], a common issue in field-collected samples from arid soils. The diversity of EMF associated with *P. cembroides* subsp. *orizabensis* is considerably low compared to other Neotropical pines [27,28,29]. The reduced number of taxa is consistent with reports for other pinyon pines, such as *Pinus edulis*, where 15 to 19 ECM taxa have been recorded in sandy-loam soils. A dominance of certain genera, such as *Geopora* and *Hebeloma*, is also observed in this type of pinyon pines, as reported by Sevanto et al. [30].

*Geopora arenicola* is reported for the first time in association with *P. cembroides* subsp. *orizabensis*. Although this EMF species has been previously documented in association with other pinyon pines such as *Pinus edulis* in drought-prone soils [31], this new record is significant, as it also occurs with another pine species inhabiting arid environments.

It is worth highlighting morphotype M9 (*Cenococcum* sp.) in transect T3, which represented the highest abundance of mycorrhizal root tips (40%). This genus is widely distributed in the Neotropics and is primarily associated with *Pinus* species. However, it has also been reported in *Pinus maximartinezii*, another pinyon pine, as a generalist symbiont. Its presence facilitates plant establishment in the field, mainly due to its drought tolerance capacity [32].

No EMF sporocarps were recorded during the study, which may be related to the low rainfall in 2023, the year when the collection was carried out, with an annual average of 221 mm [33]. Additionally, the study area experiences extreme temperatures, with highs of up to 30 °C and lows between −1 °C and −3 °C [20], reflecting high climatic variability. These factors are relevant in the context of climate change, as EMF adapted to extreme conditions are promising candidates for use as inoculants in forest nurseries aimed at reforesting eroded soils, including areas degraded by mining activities—common landscapes in the Cuenca Oriental and surrounding regions where *P. cembroides* subsp. *orizabensis* naturally occurs [3].

The survival of *P. cembroides* in arid environments may be limited in the absence of EMF, as has been observed in other *Pinus* species [34]. A reforestation study in northeastern Mexico reported survival rates of up to 50%, highlighting the significant challenges this species faces in such ecosystems [17].

Since *P. cembroides* subsp. *orizabensis* depends entirely on EMF, its survival and resilience in the face of habitat loss caused by climate change and land-use change directly depend on the availability of these fungi in the soil. The study area has been exposed to fire due to land-use change, resulting in reforested areas, primarily near agricultural fields. These changes have had a local impact on forest structure. Beta diversity analysis showed that diversity in transect T3 was largely explained by species turnover, with a higher presence of *Yucca periculosa* and fewer individuals of *P. cembroides* subsp. *orizabensis*. Additionally, this transect was recently affected by fires, which may explain the abundance of *Cenococcum* in the area, as suggested by Almaraz-Llamas et al. [32], who reported this species as abundant in fire-affected areas.

The results of this study highlight the importance of EMF in the ecology and conservation of *P. cembroides* subsp. *orizabensis*, particularly in arid and semi-arid environments. From an applied perspective, the dominance of species such as *Rhizopogon* aff. *subpurpurascens* and *Cenococcum* sp. suggests that these fungi play a key role in the adaptation and establishment of this pine under extreme conditions. Both genera are known for their drought tolerance, persistence in disturbed soils, and ability to form effective mycorrhizae under harsh conditions. *Rhizopogon* species produce long-lived spore banks and readily colonize establishing pine seedlings after disturbance, making them valuable inoculants for restoration and plantation establishment [35]. *Cenococcum* (notably *C. geophilum*) forms heavily melanized ectomycorrhizal mantles and hyphae, traits linked to desiccation resistance and persistence during drought, which can improve host water relations and survival in xeric soils [36]. Together, these genera often dominate in disturbed, sandy, or volcanic soils and thus represent promising candidates for practical inoculation strategies aimed at increasing seedling establishment, drought resilience, and long-term restoration success [37]. Additionally, the first reported association of *Geopora arenicola* with *P. cembroides* subsp. *orizabensis* expands our understanding of the diversity of mycorrhizal fungi in these ecosystems.

## 5. Conclusions

Sixteen morphotypes of EMF associated with *Pinus cembroides* subsp. *orizabensis* were identified across three transects with different cover vegetation in the Eastern Basin, Puebla. The transect with the highest *P. cembroides* coverage exhibited greater richness and evenness of morphotypes. Beta diversity analysis revealed a 60% turnover between T2 and T3, indicating distinct fungal communities, whereas between T1 and T3 a nestedness pattern without turnover was observed, suggesting a loss of diversity. Genetic identification confirmed the presence of *Tricholoma* sp., *Tomentella* sp., *Rhizopogon* aff. *subpurpurascens*, and *Geopora arenicola*. These results constitute the first report of the ectomycorrhizal community associated with this subspecies and highlight the influence of disturbance and vegetation cover on EMF composition associated with the subspecies.

## Figures and Tables

**Table 1:** Ectomycorrhizas identified through genetic sequence analysis of the ITS region, found in the roots of *Pinus cembroides* subsp. *orizabensis* from the Cuenca Oriental of Puebla.

Morphotype	Taxonomic Identification	e-Value	% Identity	Match in UNITE/Accession	e-Value	% Identity	Match in NCBI GenBank/Accession
M4	*Tomentella* sp. 1	0.0	90%	*Tomentella* sp./MW027952	0.0	91%	*Tomentella* sp./MW027950
M5	Uncultured fungus	-	-	Uncultured fungus/–	-	-	Uncultured fungus/–
M6	*Rhizopogon* aff. *subpurpurascens*	0.0	90%	*Rhizopogon subpurpurascens*/MK841952	0.0	90%	*Rhizopogon subpurpurascens*/EU669318
M7	Uncultured fungus	-	-	Uncultured fungus/–	-	-	Uncultured fungus/–
M8	*Tricholoma* sp. 1	0.0	86%	*Tricholoma moseri*/KC152252	0.0	88%	*Tricholoma moseri*/KC152252
M10	*Geopora arenicola*	0.0	97%	*Geopora arenicola*/UDB017620	0.0	98%	*Geopora arenicola*/FM206456

**Table 2:** Description of the 16 ectomycorrhizal morphotypes associated with *Pinus cembroides* subsp. *orizabensis*.

Morphotype (Taxon)	Branching Type	Color	Length (mm)	Diameter (mm)	Rhizomorphs	Emanating Hyphae	Exploration Type
M1 (*Inocybe* sp. 1)	Dichotomous, curved	Yellow	3–4	1.5	No	Rare	Short
M2 (*Pinirhiza* sp. 1)	Dichotomous, inflated, straight	Dark brown	4–5	1–1.5	No	Proximal area	–
M3 (*Pinirhiza* sp. 2)	Dichotomous, straight	Dark red	5–7	2–3	No	No	Contact
**M4 (*Tomentella* sp. 1)**	Dichotomous, cylindrical, straight	Dark brown	4–5	1	No	Distal area	Medium
**M5 (*Pinirhiza* sp. 3)**	Dichotomous, cylindrical, straight	Dark brown/black	4–6	2	Yes	Yes	Short to medium
**M6 (*Rhizopogon* aff. *subpurpurascens*)**	Dichotomous, cylindrical, curved	Light brown	4–6	1.5–2	Yes	Yes	–
M7 (*Pinirhiza* sp. 4)	Dichotomous, cylindrical, straight	Black	4–7	1.5	Yes	No	Contact
**M8 (*Tricholoma* sp. 1)**	Dichotomous, cylindrical, sinuous	Brown/golden	3–5	1.5	No	Rare	Short
M9 (*Cenococcum* sp. 1)	Dichotomous, inflated, straight	Black	3–5	2	Yes	Yes	Short
**M10 (*Geopora arenicola*)**	Dichotomous, inflated, curved	Brown/red	4	2	No	No	Medium
M11 (*Bankera* sp. 1)	Dichotomous, cylindrical, sinuous	Black	5–7	1.5	Few	No	Medium
M12 (*Russula* sp. 1)	Unbranched tips, inflated or straight	Brown	5–7	3	No	Proximal area	Contact
M13 (*Amphinema* sp. 1)	Irregular, cylindrical, straight	Dark brown	4	1.5	Few to none	Yes	Medium
M14 (*Tylospora* sp. 1)	Dichotomous, inflated, straight	Light brown to whitish	3–4	2–3	No	No	Short
M15 (Russulaceae)	Dichotomous, turgid, straight	Light brown	4–5	1	No	No	Contact
M16 (*Paxillus* sp. 1)	Dichotomous, cylindrical, straight	Whitish	4–6	2	No	No	Long

Taxa in bold were identified using the ITS region, while the remaining ones were identified morphologically.

## Data Availability

The original contributions presented in this study are included in the article. Further inquiries can be directed to the corresponding authors.

## Abbreviations

The following abbreviations are used in this manuscript:

EMF	Ectomycorrhizal fungi
DNA	Deoxyribonucleic Acid
